# Association of simultaneously measured four-limb blood pressures with cardiovascular function: a cross-sectional study

**DOI:** 10.1186/s12938-016-0266-y

**Published:** 2016-12-28

**Authors:** Xiaorui Song, Gaoyang Li, Aike Qiao, Zhihui Chen

**Affiliations:** 1 College of Life Science and Bioengineering, Beijing University of Technology, No.100, Pingleyuan, Chaoyang District, Beijing, China; 2 School Hospital, Beijing University of Technology, No.100, Pingleyuan, Chaoyang District, Beijing, China

**Keywords:** Cardiovascular function, Four limbs, Blood pressure difference, Simultaneous measurement

## Abstract

**Background:**

Simultaneous measurement of four-limb blood pressures can improve the accuracy of cardiovascular disease diagnosis. This study aims to investigate the association of simultaneously measured four-limb blood pressures with cardiovascular function as the non-invasive diagnostic method of cardiovascular disease in primary care.

**Methods:**

229 subjects (62 males, mean age, 63.50 ± 11.13 years; 167 females, mean age, 59.47 ± 7.33 years) were enrolled. Four-limb blood pressure measurements were simultaneously performed using a blood pressure and pulse monitor device in the supine position. Cardiac functional parameters were also measured by using a cardiac hemodynamic detector in the same position. Data were statistically analyzed with SPSS15.0.

**Results:**

The mean age of the 229 subjects was 60.56 ± 8.68 years. Cardiovascular functional parameters decreased with age and body mass index (BMI), only the total peripheral resistance (TPR) was in contrast. Age, BMI, left ankle diastolic pressure (LADP), high arm mean arterial pressure (HARMAP), left arm diastolic pressure (LARDP) and right ankle diastolic pressure (RADP) were significantly correlated with cardiovascular functional parameters. Cardiovascular functional parameters have significant differences with inter-arm difference in systolic blood pressure (SBP) between ≥10 and <10 mmHg, inter-ankle difference in SBP between ≥15 and ≥20 mmHg, inter-ankle difference in SBP between ≥15 and <10 mmHg and right ankle brachial index (RABI) between ≤0.9 and ≥1.0. After excluding 99 hypertensive patients, a part of cardiovascular functional parameters has still significant differences with inter-arm difference in SBP between ≥10 and ≥15 mmHg and RABI between ≤0.9 and ≥1.0.

**Conclusion:**

Age, BMI, LADP, HARMAP, LARDP and RADP were the determinants of cardiovascular functional parameters. In addition, a part of cardiovascular functional parameter is associated with inter-arm difference in SBP ≥10 mmHg, inter-ankle difference in SBP ≥15 mmHg and RABI ≤0.9, while these differences still existed after excluding 99 hypertensive patients. Hence, simultaneous measurement of four-limb blood pressures has become feasible and useful approach to the non-invasive diagnostic method of cardiovascular disease in primary care.

## Background

Accurate measurement of blood pressure and scientific evaluation are the precondition for the early detection of cardiovascular disease. The studies found that four-limb blood pressure simultaneous measurement can improve the accuracy of blood pressure for cardiovascular disease diagnosis [1–3]. Therefore, it is an important that four-limb blood pressure should be simultaneously measured to identify and manage the cardiovascular disease. However, most evidences on cardiovascular disease from these studies are obtained by either measuring single limb blood pressure or performing sequence measurement instead of simultaneous four limbs measurement [4–6]. Current technology has allowed to measure four-limb blood pressure simultaneously [7], which could generate accurate blood pressure differences between four limbs, provide a comprehensive evaluation of blood pressure and improve the accuracy of blood pressure for cardiovascular disease diagnosis [2, 4].

A blood pressure difference between arms has been associated with subclavian stenosis, peripheral artery disease, cardiovascular mortality and all-cause mortality [1, 8–11], meanwhile recent studies on inter-leg systolic blood pressure difference have added a new evidence to this concept [12–14]. The meta-analysis reported by Cao showed that inter-arm systolic blood pressure difference ≥15 mmHg might help to predict increased cardiovascular mortality (HR 1.94, 95% CI 1.12–3.35, P < 0.05) in the community populations [15]. However, the other meta-analysis reported by Singh showed that there was not statistically direct association of cardiovascular mortality with inter-arm systolic blood pressure difference of 10 mmHg or more (OR 1.82; CI 0.68–4.88; P = 0.23), 15 mmHg or more (OR 1.66; CI 0.68–4.07; P = 0.27), and inter-leg systolic blood pressure difference of 15 mmHg or more (OR 1.97; CI 0.72–5.34; P = 0.19) [2]. Although the importance of blood pressure difference between arms or between legs is sometimes already recognized [1, 8–14], association of four limbs blood pressure differences with cardiovascular mortality and morbidity remains controversial.

Accordingly, this study aims to investigate the association of simultaneously measured four-limb blood pressures with cardiovascular function as the current non-invasive diagnostic method of cardiovascular disease in primary care.

## Methods

### Subjects

This study was approved by the Ethics Committee of Hospital in Beijing University of Technology, and College of Life Science and Bioengineering in Beijing University of Technology. All subjects gave written informed consent. From September 2015 to January 2016, staffs of Beijing University of Technology took part in comprehensive examinations of cardiovascular disease and its risk evaluation. Subjects with limb disability, hemiplegia, congenital heart disease, heart failure, and the history of artery intervention were excluded. Finally, 229 subjects (62 males, mean age, 63.50 ± 11.13 years; 167 females, mean age, 59.47 ± 7.33 years) were enrolled in this study.

### Four-limb blood pressure measurements

Four-limb blood pressure was measured in an air-conditioned room at a temperature of 22–23 °C by using the VS-1500 blood pressure and pulse monitor device (Fukuda Company, Beijing, China). Trained technicians placed the blood pressure cuffs on both arms and both ankles and performed the measurements, after each subject had bared four limbs and taken 10-min rest in supine position. The device simultaneously and automatically measured the supine blood pressure of four limbs, and automatically calculated the ankle-brachial index (ABI) [ABI include right ankle-brachial index (RABI) and left ankle-brachial index (LABI)], and then stored the measurement data in a database.

Based on the systolic and diastolic blood pressure, we calculated the inter-arm and inter-ankle blood pressure differences as the absolute value of the difference between the right and left arm blood pressure and between the right and left ankle blood pressure, respectively. Pulse pressure (PP) was the absolute value of difference between systolic and diastolic blood pressure. Pulse pressure index (PPI) was calculated as the ratio of PP divided by systolic blood pressure. Mean arterial pressure (MAP) was two-thirds diastolic pressure plus one-third systolic pressure.

### Cardiovascular function measurements

**Fig. 1:**
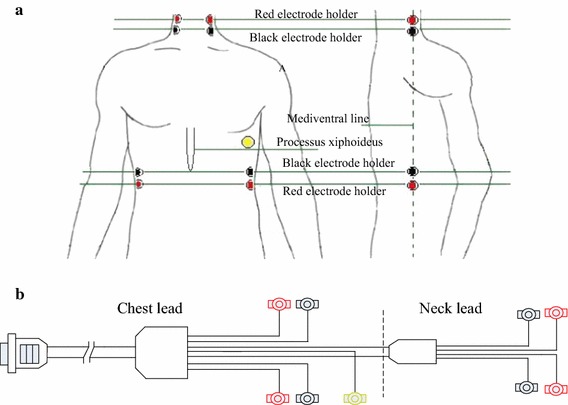
Specific measurement positions of cardiovascular function parameters. **a** Measurement positions of electrode holder. **b** Electrode holder measuring device

Cardiac functional parameters were also measured by using a cardiac hemodynamic detector device (Boundless Horizon Company, Shandong, China) in the supine position and in the same air-conditioned room after obtaining four limbs blood pressure. Before cardiovascular function measurement, each subject had bared abdomen and neck, stuck to electrode slices and taken 10-min rest in supine position. Trained technicians placed the red/black electrode holders on abdomen and neck, placed one yellow electrode holder on the fifth rib left anterior axillary line, respectively. The specific measurement positions were shown in Fig. 1. The device automatically measured the cardiac functional parameters, and then stored the measurement data in a database. Cardiovascular functional parameters related mainly to five parameters in this study: cardiac pump function, cardiac systolic, cardiac diastolic, cardiac efficiency and vascular elasticity.

Cardiac pump function parameters include cardiac output per minute (CO), stroke volume (SV), cardiac index (CI), stroke volume index (SVI) and ejection fraction (EF). Cardiac systolic function parameters have function index of left ventricular (LFVI), index of contractility (IC) and heather index (HI). Cardiac diastolic function parameters have end diastolic volume (EDV) and left ventricular end diastolic pressure (LVEDP). Cardiac efficiency function parameters have stroke work (SW), cardiac work (CW), stroke work index (SWI) and cardiac work index (CWI). Vascular elasticity function parameters have aortic compliance (AC) and total peripheral resistance (TPR).

In addition, the observer also administered a standardized questionnaire to collect information of subjects on age, sex, height, weight, medical history, lifestyle, use of medications, drinking and smoking history. The body mass index (BMI) was calculated as the ratio of weight in kilograms divided by the square of height in meters.

### Statistical analysis

Data were stored in Excel 2013 and statistically analyzed with SPSS15.0. Data were expressed as percentages and mean ± SD. The differences of inter-arm and inter-ankle were divide into five groups (<5, 5–9, 10–14, 15–19 and ≥20), and ABI were divide into three groups (≤0.9, 0.91–0.99, ≥1.0). The differences between groups were checked by the analysis of variance for continuous variables or by Chi square test for categorical variables. Pearson correlation analysis was used to determine the correlation degree between cardiac functional parameters and four-limb blood pressure. Multiple linear regression analysis was used to determine the relationship between cardiac functional parameters and four-limb blood pressure. A difference was considered significant if the P value was less than 0.05.

## Results

### Baseline characteristics of subjects

The mean age of the 229 subjects was 60.56 ± 8.68 years, 9 subjects were younger than 45 years, 31 subjects between 45 and 54 years, 136 subjects between 55 and 64 years, 39 subjects between 65 and 74 years, and 14 subjects were aged 75 years or older. Table 1 presents the clinical characteristics of the subjects by gender. The cardiovascular functional parameter difference in CI, EF, LFVI, IC, HI, EDV was significance between male and female (P < 0.05). Also, independent-samples T test was performed between hypertension patients and the general population, and it was found that the differences of cardiovascular functional parameters as CI, SV, CO, SVI, EF, LFVI, IC, HI, LVEDP, EDV, AC and TPR were significant (P < 0.05) between them. The mean values of these parameters in hypertension patients were lower than those of the general population except TPR.

**Table 1:** Baseline characteristics of the study participants

Characteristics		Male (n = 62)	Female (n = 167)	P
Age, years		63.50 ± 11.13	59.47 ± 7.33	0.002
Body mass index, kg/m^2^		24.54 ± 3.15	25.75 ± 3.70	0.023
Simultaneous four-limb BP measurement, mmHg				
Left arm	Systolic pressure (LARSP)	136.69 ± 19.01	135.78 ± 18.22	0.740
	Diastolic pressure (LARDP)	82.50 ± 10.96	81.02 ± 10.28	0.342
Right arm	Systolic pressure (RARSP)	136.89 ± 18.47	135.78 ± 18.58	0.688
	Diastolic pressure (RARDP)	83.21 ± 11.87	81.32 ± 10.06	0.230
Left ankle	Systolic pressure (LASP)	151.48 ± 26.19	150.38 ± 22.77	0.754
	Diastolic pressure (LADP)	79.82 ± 10.87	77.25 ± 8.30	0.057
Right ankle	Systolic pressure (RASP)	150.45 ± 25.93	150.47 ± 22.78	0.997
	Diastolic pressure (RADP)	77.05 ± 11.50	75.16 ± 7.58	0.150
BP on the higher arm/ankle side of systolic pressure, mmHg				
Arm	Systolic pressure (HARSP)	140.27 ± 18.79	139.03 ± 18.44	0.652
	Diastolic pressure (HARDP)	83.94 ± 11.56	81.72 ± 10.38	0.166
	Pulse pressure (HARPP)	56.34 ± 14.19	57.31 ± 10.38	0.647
	Pulse pressure index (HARPPI)	0.40 ± 0.06	0.41 ± 0.06	0.299
	Mean arterial pressure (HARMAP)	102.72 ± 12.73	100.03 ± 11.85	0.295
Ankle	Systolic pressure (HASP)	154.50 ± 25.99	153.79 ± 22.51	0.839
	Diastolic pressure (HADP)	80.02 ± 11.81	77.11 ± 8.50	0.041
	Pulse pressure (HAPP)	74.48 ± 18.27	76.68 ± 17.93	0.414
	Pulse pressure index (HAPPI)	0.48 ± 0.07	0.49 ± 0.06	0.046
Inter-arm BP difference, mmHg				
Systolic pressure	mean ± SD	6.97 ± 7.66	6.63 ± 5.98	0.725
Diastolic pressure	mean ± SD	4.35 ± 3.30	3.95 ± 3.84	0.466
Systolic pressure	≥10 mmHg, n (%)	9 (14.5)	23 (13.7)	0.705
Systolic pressure	≥15 mmHg, n (%)	9 (14.5)	17 (10.2)	0.528
Diastolic pressure	≥10 mmHg, n (%)	3 (4.8)	7 (4.1)	0.362
Diastolic pressure	≥15 mmHg, n (%)	1 (1.6)	4 (2.4)	0.702
Inter-ankle BP difference, mmHg				
Systolic pressure	mean ± SD	7.06 ± 7.87	6.74 ± 5.20	0.715
Diastolic pressure	mean ± SD	4.87 ± 4.07	4.11 ± 3.48	0.165
Systolic pressure	≥10 mmHg, n (%)	5 (8.1)	26 (15.6)	0.712
Systolic pressure	≥15 mmHg, n (%)	9 (14.5)	16 (9.6)	0.001
Diastolic pressure	≥10 mmHg, n (%)	4 (6.5)	18 (10.8)	0.054
Diastolic pressure	≥15 mmHg, n (%)	3 (4.8)	0 (0)	–
Arm-ankle BP difference, mmHg				
L-ABI	mean ± SD	1.08 ± 0.15	1.08 ± 0.10	0.979
R-ABI	mean ± SD	1.08 ± 0.16	1.08 ± 0.09	0.734
L-ABI	≤0.9, n (%)	4 (6.1)	6 (3.6)	0.015
	0.91–0.99, n (%)	6 (9.7)	30 (18.0)	0.852
	≥1.00, n (%)	52 (83.9)	131 (78.4)	0.497
R-ABI	≤0.9, n (%)	5 (8.1)	5 (3.0)	0.176
	0.91–0.99, n (%)	7 (11.3)	26 (15.6)	0.992
	≥1.00, n (%)	50 (80.6)	136 (81.4)	0.251
Cardiac pump function				
CO		5.27 ± 1.16	5.26 ± 1.19	0.969
SV		78.90 ± 17.98	76.81 ± 18.19	0.439
CI		2.98 ± 0.76	3.23 ± 0.82	0.034
SVI		44.69 ± 11.84	47.34 ± 12.63	0.152
EF		0.64 ± 0.07	0.69 ± 0.07	0.000
Cardiac systolic function				
LFVI		0.17 ± 0.02	0.16 ± 0.01	0.002
IC		0.05 ± 0.01	0.06 ± 0.02	0.000
HI		17.02 ± 4.84	22.03 ± 6.83	0.000
Cardiac diastolic function				
EDV		121.41 ± 17.57	110.23 ± 18.14	0.000
LVEDP		10.30 ± 3.41	9.46 ± 2.98	0.067
Cardiac efficiency				
SW		0.11 ± 0.02	0.10 ± 0.02	0.140
CW		7.09 ± 1.55	6.93 ± 1.67	0.511
SWI		0.06 ± 0.01	0.06 ± 0.02	0.303
CWI		4.00 ± 0.96	4.26 ± 1.12	0.109
Vascular elasticity				
AC		1.70 ± 0.60	1.74 ± 0.83	0.705
TPR		1598.87 ± 453.21	1558.99 ± 408.93	0.525

### Pearson correlation analysis between four-limb blood pressures and cardiovascular functional parameters

Pearson correlation analysis presents the correlation degree between four-limb blood pressures and cardiovascular functional parameters as shown in Table 1. Cardiac pump function parameters (CO, SV, CI, SVI, and EF) have significant differences (P < 0.05) and a negative correlation with age, BMI, HARSP, HARDP, HARPP, HARMAP, HASP, HADP, HAPP, RARSP, RARDP, LARSP, LARDP, RASP, RADP, LASP, and LADP. Cardiac systolic function parameters (IC and HI) have significant differences (P < 0.05) and a negative correlation with age, BMI, HARDP, HARMAP, HASP, HADP, LARDP, RADP, LASP, and LADP. Cardiac diastolic function parameters (EDV and LVEDP) have significant differences (P < 0.05) and a negative correlation with BMI, HARSP, HARDP, HARMAP, HASP, HADP, HAPP, RARSP, RARDP, LARSP, LARDP, RASP, RADP, LASP, and LADP. Cardiac efficiency function parameters (SW, CW, SWI, and CWI) have significant differences (P < 0.05) and a negative correlation with age and BMI, SWI also has significant differences (P < 0.05) and a negative correlation with HARSP, HARDP, HARMAP, HASP, HADP, RARDP, LARSP, LARDP, RASP, RADP, LASP, and LADP. Vascular elasticity function parameter (AC and TPR) have significant differences (P < 0.05) with age, BMI, HARSP, HARDP, HARPP, HARMAP, HASP, HADP, HAPP, RARSP, RARDP, LARSP, LARDP, RASP, RADP, LASP, and LADP, AC has a negative correlation with these parameters, and TPR has a position correlation with these parameters. Cardiovascular functional parameters have no significant differences with inter-arm blood pressure difference, inter-ankle blood pressure difference and ABI.

### Multiple linear regression analysis between four-limb blood pressures and cardiovascular functional parameters

Multiple linear regression stepwise analysis presents the determinants of cardiovascular functional parameter as shown in Table 2. The independent factors negatively correlated with CO were found to be age, BMI and LADP (β = −0.250, −0.332, −0.190; all P < 0.05). The independent factors negatively correlated with SV were found to be age, BMI, LADP and HARMAP (β = −0.250, −0.318, −0.186, −0.157; all P < 0.05). The independent factors negatively correlated with CI were found to be age, BMI and LADP (β = −0.240, −0.482, −0.204; all P < 0.05). The independent factors negatively correlated with SVI were found to be age, BMI and LARDP (β = −0.255, −0.457, −0.185; all P < 0.05). The independent factors negatively correlated with EF were found to be age, BMI, LARDP and LADP (β = −0.297, −0.252, −0.173, −0.216; all P < 0.05). The independent factors negatively and positively correlated with LFVI were found to be HARPP (β = −0.280; P < 0.05) and age (β = 0.186; P < 0.05) respectively. The independent factors negatively and positively correlated with IC were found to be age, BMI (β = −0.313, −0.290; all P < 0.05) and HARPPI (β = 0.148; P < 0.05) respectively. The independent factors negatively and positively correlated with HI were found to be age, BMI, LADP (β = −0.363, −0.230, −0.255; all P < 0.05) and HARPPI (β = 0.161; P < 0.05) respectively. The independent factors negatively correlated with EDV were found to be age, BMI and HARMAP (β = −0.148, −0.308, −0.240; all P < 0.05). The independent factors negatively and positively correlated with LVEDP were found to be BMI, LASP (β = −0.198, −0.224; all P < 0.05) and ADBPD (β = 0.131; P < 0.05) respectively. The independent factors negatively correlated with SW were found to be age and BMI (β = −0.203, −0.344; all P < 0.05). The independent factors negatively and positively correlated with CW were found to be age, BMI, LADP (β = −0.178, −0.313, −0.194; all P < 0.05) and ADBPD (β = 0.131; P < 0.05) respectively. The independent factors negatively correlated with SWI were found to be age and BMI (β = −0.201, −0.504; all P < 0.05). The independent factors negatively and positively correlated with CWI were found to be age, BMI, LADP (β = −0.211, −0.484, −0.242; all P < 0.05) and RARSP, RADP (β = 0.184, 0.209; P < 0.05) respectively. The independent factors negatively correlated with AC were found to be age, BMI and LARSP (β = −0.167, −0.175, −0.434; all P < 0.05). The independent factors positively correlated with TPR were found to be age, BMI, LADP and HARMAP (β = 0.238, 0.296, 0.191, 0.260; all P < 0.05).

**Table 2:** Multiple linear regression stepwise analysis between four-limb blood pressure and cardiovascular functional parameters

Dependent variable	R	Variable	β	P	95% CI
CO	0.500	Age	−0.250	0.000	(−0.050 to −0.018)
		BMI	−0.332	0.000	(−0.148 to −0.070)
		LADP	−0.190	0.002	(−0.040 to −0.009)
SV	0.586	Age	−0.250	0.000	(−0.749 to −0.292)
		BMI	−0.318	0.000	(−2.161 to −1.044)
		HARMAP	−0.157	0.046	(−0.465 to −0.004)
		LADP	−0.186	0.016	(−0.670 to −0.070)
CI	0.625	Age	−0.240	0.000	(−0.032 to −0.013)
		BMI	−0.482	0.000	(−0.132 to −0.085)
		LADP	−0.204	0.000	(−0.028 to −0.009)
SVI	0.688	Age	−0.255	0.000	(−0.504 to −0.288)
		BMI	−0.457	0.000	(−1.925 to −1.240)
		LARDP	−0.185	0.006	(−0.377 to −0.063)
		LADP	−0.169	0.013	(−0.411 to −0.050)
EF	0.591	Age	−0.297	0.000	(−0.003 to −0.002)
		BMI	−0.252	0.000	(−0.007 to −0.003)
		LARDP	−0.173	0.021	(−0.002 to 0.000)
		LADP	−0.216	0.004	(−0.003 to −0.001)
LFVI	0.294	Age	0.186	0.005	(0.000 to 0.001)
		HARPP	−0.280	0.000	(0.000 to 0.000)
IC	0.430	Age	−0.313	0.000	(−0.001 to 0.000)
		BMI	−0.290	0.000	(−0.002 to −0.001)
		HARPPI	0.148	0.016	(0.007 to 0.071)
HI	0.554	Age	−0.363	0.000	(−0.367 to −0.194)
		BMI	−0.230	0.000	(−0.643 to −0.218)
		HARPPI	0.161	0.005	(5.143 to 28.907)
		LADP	−0.255	0.000	(−0.273 to 0.0104)
EDV	0.475	Age	−0.148	0.014	(−0.572 to −0.064)
		BMI	−0.308	0.000	(−2.215 to −0.979)
		HARMAP	−0.240	0.000	(−0.557 to −0.181)
LVEDP	0.327	BMI	−0.198	0.003	(−0.283 to −0.061)
		LASP	−0.224	0.001	(−0.046 to −0.013)
		ADBPD	0.131	0.044	(0.003 to 0.220)
SW	0.400	Age	−0.203	0.001	(−0.001 to 0.000)
		BMI	−0.344	0.000	(−0.003 to −0.001)
CW	0.415	Age	−0.178	0.004	(−0.056 to −0.011)
		BMI	−0.313	0.000	(−0.199 to −0.086)
		RARDP	0.347	0.000	(0.028 to 0.080)
		LADP	−0.194	0.026	(−0.065 to −0.004)
SWI	0.544	Age	−0.201	0.000	(−0.001 to 0.000)
		BMI	−0.504	0.000	(−0.003 to −0.002)
CWI	0.547	Age	−0.211	0.000	(−0.041 to −0.012)
		BMI	−0.484	0.000	(−0.180 to −0.111)
		RARSP	0.184	0.027	(0.001 to 0.020)
		LADP	−0.242	0.003	(−0.048 to −0.010)
		RADP	0.209	0.020	(0.003 to 0.039)
AC	0.562	Age	−0.167	0.004	(−0.025 to −0.005)
		BMI	−0.175	0.002	(−0.062 to −0.014)
		LARSP	−0.434	0.000	(−0.023 to −0.005)
TPR	0.644	Age	0.238	0.000	(6.506 to 16.539)
		BMI	0.296	0.000	(22.357 to 46.836)
		HARMAP	0.191	0.001	(1.587 to 11.696)
		LADP	0.260	0.000	(5.432 to 18.592)

### Variance analysis between four-limb blood pressure differences and cardiovascular functional parameters

**Fig. 2:**
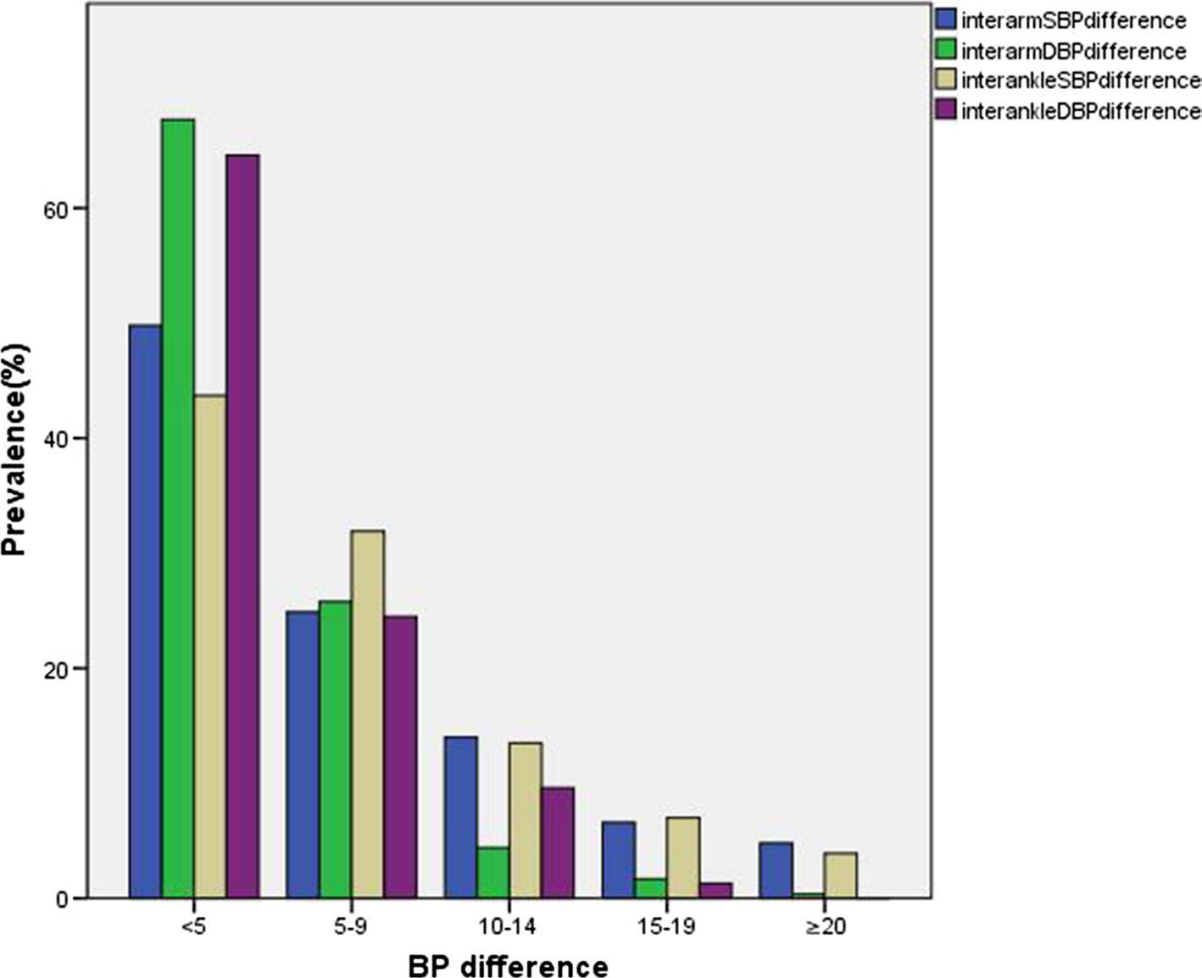
The distribution of four-limb blood pressure difference in the subjects

**Fig. 3:**
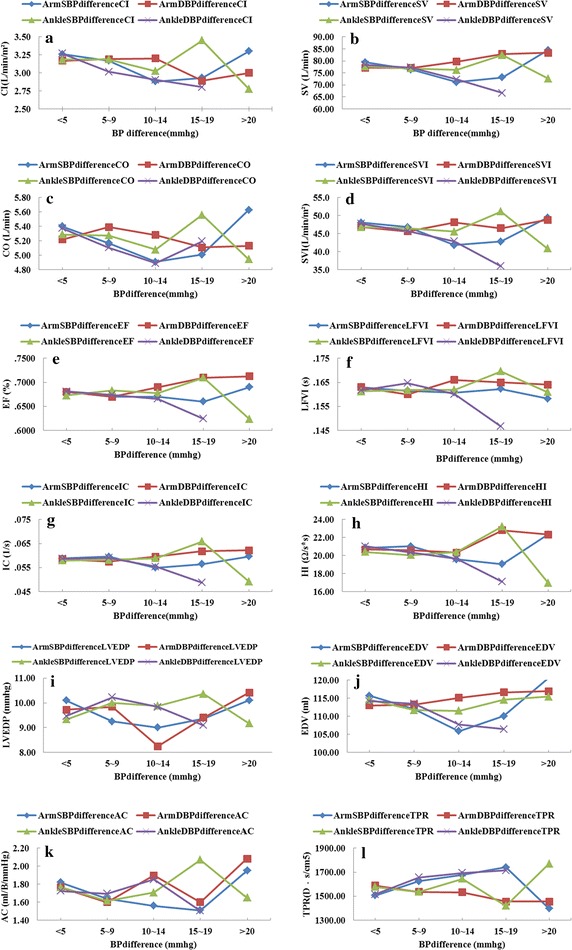
The mean distribution of cardiovascular functional parameters among four-limb blood pressure difference in this study subjects. **a** Mean distribution of CI among four-limb blood pressure differences. **b** Mean distribution of SV among four-limb blood pressure differences. **c** Mean distribution of CO among four-limb blood pressure differences. **d** Mean distribution of SVI among four-limb blood pressure differences. **e** Mean distribution of EF among four-limb blood pressure differences. **f** Mean distribution of LFVI among four-limb blood pressure differences. **g** Mean distribution of IC among four-limb blood pressure differences. **h** Mean distribution of HI among four-limb blood pressure differences. **i** Mean distribution of LVEDP among four-limb blood pressure differences. **j** Mean distribution of EDV among four-limb blood pressure differences. **k** Mean distribution of AC among four-limb blood pressure differences. **l** Mean distribution of TPR among four-limb blood pressure differences

**Fig. 4:**
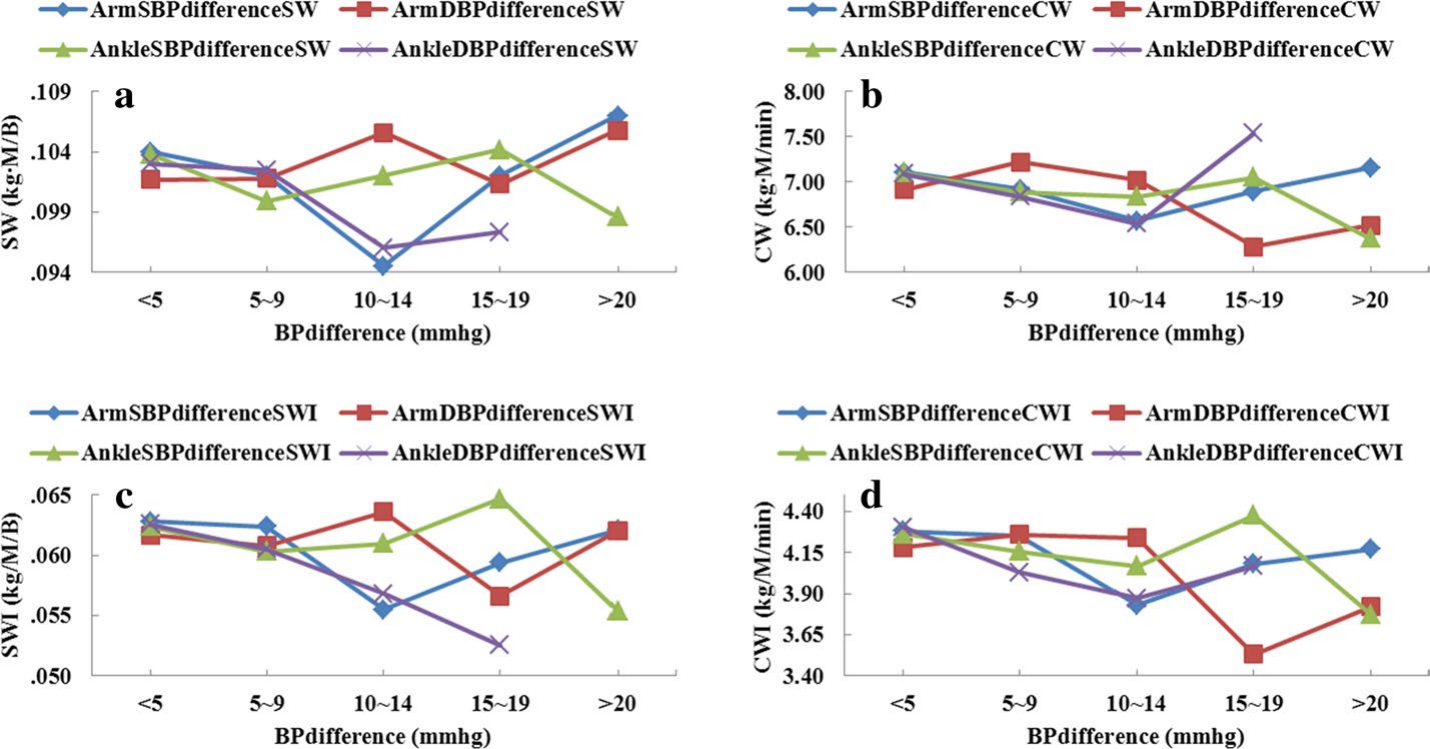
The mean distribution of cardiovascular functional parameters among four-limb blood pressure difference in this study subjects. **a** Mean distribution of SW among four-limb blood pressure differences. **b** Mean distribution of CW among four-limb blood pressure differences. **c** Mean distribution of SWI among four-limb blood pressure differences. **d** Mean distribution of CWI among four-limb blood pressure differences

In additional, variance analysis was performed to check the difference among the cardiovascular functional parameters, four-limb blood pressure differences (<5, 5–9, 10–14, 15–19 and ≥20) and ABI (≤0.9, 0.91–0.99, ≥1.0). The distribution of four-limb blood pressure differences as shown in Fig. 2. There were 11.35, 4.37, 10.92, and 9.61% of subjects with an inter-arm difference in systolic blood pressure of >15 mmHg, inter-arm difference in diastolic blood pressure of >10 mmHg, inter-ankle difference in systolic blood pressure of >15 mmHg and inter-ankle difference in diastolic blood pressure of >10 mmHg, respectively. The mean distribution of cardiovascular functional parameters among four-limb blood pressure difference (<5, 5–9, 10–14, 15–19 and ≥20) as shown in Figs. 3 and 4. Cardiovascular functional parameters (CI, SV, CO, SVI, EDV, TPR, SW, SWI and CWI) have significant differences (P < 0.05) with inter-arm difference in systolic blood pressure between ≥10 and <10 mmHg. Cardiovascular functional parameters (EF, HI and TPR) have significant differences (P < 0.05) with inter-ankle difference in systolic blood pressure between ≥15 and ≥20 mmHg. Cardiovascular functional parameters (LFVI and AC) have significant differences (P < 0.05) with inter-ankle difference in systolic blood pressure between ≥15 and <10 mmHg. CI, CO, LVEDP, CW, CWI have significant differences (P < 0.05) with RABI between ≤0.9 and ≥1.0.

### Analysis between four-limb blood pressures and cardiovascular functional parameters in subgroup

Since cardiovascular function has been associated with hypertension, we also performed a subgroup analysis after excluding 99 hypertension patients. After Pearson correlation analysis in subgroup, we found that a part of blood pressure parameters correlation with cardiovascular functional parameters disappeared, and the correlation coefficient reduced. In addition, after analysis of variance in subgroup, we also found that cardiovascular functional parameters (SV, EDV and SW) have significant differences (P < 0.05) with inter-arm difference in systolic blood pressure between ≥10 and ≥15 mmHg, and cardiovascular functional parameters (CI, CO, TPR, CWI and HI) have significant differences (P < 0.05) with RABI between ≤0.9 and ≥1.0.

## Discussion

In this cross-sectional study, by using a simultaneous measurement technique, the association between four-limb blood pressure and cardiovascular function and related risk factors was evaluated. Cardiovascular functional parameters illustrated the cardiac pump function, cardiac systolic, cardiac diastolic, cardiac efficiency and vascular elasticity. Thus, this study has more systematicness and pertinence than previous studies. This study suggested that cardiovascular functional parameters (CO, SV, CI, SVI, EF, IC, HI, EDV, LVEDP, SWI, AC and TPR) have all a significant difference with HARDP, HARMAP, HASP, HADP, LARDP, RADP, LASP, and LADP, while there was a negative correlation between them except TPR. Meanwhile, the determinants of cardiac functional parameter were age, BMI, LADP, HARMAP LARDP, HARPPI ADBPD, RARSP, RADP and LARSP, while the TPR was positively correlated with age, BMI, LADP and HARMAP.

Previous studies demonstrated that population aging has become the uncontrolled risk factor of cardiovascular disease [16–18]. It is a physiology problem that the effect between blood pressure and cardiovascular function is mutual. The cardiovascular function decreased gradually with age, especially arteries can produce physiological degeneration. The thickening of vessels wall, the decrease or even rupture and calcification of elastic fibers, the increase of peripheral resistance, the decrease of blood vessel compliance, all these factors will result in the systolic blood pressure and diastolic blood pressure increasing with age. In addition, it is a fluid mechanics problem that different arteries away from heart have different resistance and pressure. According to the Poiseuille’s law, brachial artery resistance is relatively small and the blood pressure is low because heart is close to the upper limb, while the phenomenon of the ankle artery is in contrast. Hence blood pressures in four limbs are different. In addition, the distance from the heart affects pressure wave propagation and reflection because of the geometry tapper and elasticity tapper, and augments the pressure of peripheral vascular compared with that of upper limb.

Previous studies have reported that inter-arm or inter-ankle blood pressure difference predicted cardiovascular mortality [1, 2, 8–13, 15]. These results suggested that the simultaneous measurement of four-limb blood pressure is needed to improve diagnostic accuracy between cardiovascular disease and blood pressure difference [2–5]. The present study demonstrated the significant correlation between cardiovascular functional parameters and four-limb blood pressure, while cardiovascular functional parameters had no significant differences with inter-arm blood pressure difference, inter-ankle blood pressure difference and ABI. However, the mean plots of cardiovascular functional parameters present obvious change trend in four-limb systolic blood pressure difference and diastolic blood pressure difference ≥10 or ≥15 mmHg. These results were different form previous studies. Verberk et al. [19] reported that the prevalence of inter-arm difference in systolic blood pressure of 10 mmHg or more was roughly doubled when diagnosis measurements method used a sequential approach, or used manual approach rather than automated measurements approach. Thus, simultaneous measurement of four-limb blood pressure and calculation of four-limb blood pressure difference may be helpful and necessary in evaluating the heart function and predicting patients with cardiovascular disease. Further study, the subgroup analysis showed that the predictive value of four limbs blood pressure for cardiovascular function. Subgroup analysis found that a part of cardiovascular functional parameter has still significant differences (P < 0.05) with inter-arm difference in systolic blood pressure between ≥10 and ≥15 mmHg, and with RABI between ≤0.9 and ≥1.0. However, the exact reason is unclear now, hypertension is the influence factors of cardiovascular function parameters, and four-limb blood pressure differences are also applicable to diagnose hypertensive patients. As mentioned above, four limbs blood pressures were not only influence factors of cardiovascular function parameters, but a predictor for cardiovascular disease, evaluating cardiovascular function parameters was important in clinical practice and epidemiological studies. In addition, the better way for cardiovascular function parameters was simultaneous blood pressures for four limbs.

A clinical device, the VS-1500 blood pressure and pulse monitor device (Fukuda Company, Beijing, China), has been developed to automatically and simultaneously measure blood pressure in four limbs, and the measurement can be easily obtained. The similar device was reported by previous studies, for example, VP-1000 ABI—form device (Colin Co. Ltd., Komaki, Japan) and VP-1000 device (Omron, Kyoto, Japan) were used to automatically and simultaneously measure four limbs’ blood pressures. The validation in measurement technology of device (Boundless Horizon Company, Shandong, China) might be prone for measurement errors, especially in multinomial detection of cardiac function parameters rather than single detection. Although it could not give very precise measurements for the clinical value of cardiac function parameters, it can provide objective evaluation of health in the early stage of cardiovascular diseases. Thus, these devices can be applied for the epidemiological evaluation between four limbs’ blood pressures and cardiac function parameters.

In this study, by using a simultaneous and noninvasive measurement technique, we measured four limbs blood pressures. But this technique was not a popular one to measure blood pressure in daily clinical practice. Hence, although four limbs blood pressure with simultaneous measurement could improve the predictive value for cardiovascular disease, our results might be changed if daily clinical measurement was used. In addition, the subjects were mainly from retired people, whose health care consciousness is better than the serving officer. The factors of smoking, drinking, salting and movement have no significant difference in this study subjects. Hence, the clinical utility of this study may be limited in community people.

## Conclusion

LADP, HARMAP, LARDP, and RADP were all significantly correlated with cardiovascular functional parameter. Age and body mass index are all the major risk factors for cardiovascular function parameters. In addition, a part of cardiovascular functional parameter is associated with inter-arm difference in systolic blood ≥10 mmHg, inter-ankle difference in systolic blood pressure ≥15 mmHg and RABI ≤0.9, while these differences still exist after excluding 99 hypertension patients. Hence, simultaneous measurement of four-limb blood pressures has become feasible and useful approach to the current non-invasive method of cardiovascular disease in primary care.

## Abbreviations

CO: cardiac output per minute
CI: cardiac index
SV: stroke volume
SVI: stroke volume index
EF: ejection fraction
LFVI: function index of left ventricular
IC: index of contractility
HI: heather index
EDV: end diastolic volume
SW: stroke work
CW: cardiac work
SWI: stroke work index
CWI: cardiac work index
AC: aortic compliance
TPR: total peripheral resistance
HR: heart rate
SBP: systolic blood pressure
LARSP: left arm systolic pressure
LARDP: left arm diastolic pressure
RARSP: right arm systolic pressure
RARDP: right arm diastolic pressure
LASP: left ankle systolic pressure
LVEDP: left ventricular end diastolic pressure
LADP: left ankle diastolic pressure
RASP: right ankle systolic pressure
RADP: right ankle diastolic pressure
HARSP: high arm systolic pressure
HARDP: high arm diastolic pressure
HARPP: high arm pulse pressure
HARPPI: high arm pulse pressure index
HASP: high ankle systolic pressure
HADP: high ankle diastolic pressure
HAPP: high ankle pulse pressure
HAPPI: high ankle pulse pressure index
ABI: ankle brachial index
RABI: right ankle brachial index
LABI: left ankle brachial index
PP: pulse pressure
PPI: pulse pressure index
MAP: mean arterial pressure
BMI: body mass index
HARMA: high arm mean arterial pressure
